# Cyst infection in autosomal dominant polycystic kidney disease: penetration of meropenem into infected cysts

**DOI:** 10.1186/s12882-018-1067-2

**Published:** 2018-10-19

**Authors:** Satoshi Hamanoue, Tatsuya Suwabe, Yoshifumi Ubara, Koichi Kikuchi, Ryo Hazue, Koki Mise, Toshiharu Ueno, Kenmei Takaichi, Kana Matsumoto, Kunihiko Morita

**Affiliations:** 1 Department of Nephrology, Toranomon Hospital Kajigaya, Kawasaki, Japan; 2 Okinaka Memorial Institute for Medical Research, Toranomon Hospital, Tokyo, Japan; 3 Department of Clinical Pharmaceutics, Faculty of Pharmaceutical Sciences, Doshisha Women’s College of Liberal Arts, Kyoto, Japan

**Keywords:** ADPKD, Carbapenem, Cyst infection, Meropenem, Infected cyst, Polycystic kidney disease

## Abstract

**Background:**

Cyst infection is a common and serious complication of autosomal dominant polycystic kidney disease (ADPKD) that is often refractory. Carbapenems are frequently needed to treat to patients with refractory cyst infection, but little is known about the penetration of newer water-soluble carbapenems into cysts. This study investigated the penetration of meropenem (MEPM) into infected cysts in patients with ADPKD.

**Methods:**

Between August 2013 and January 2014, 10 ADPKD patients (14 infected cysts) receiving MEPM at Toranomon Hospital underwent drainage of infected cysts and definite cyst infection was confirmed through detection of neutrophils by cyst fluid analysis. The serum concentration of MEPM was measured just after intravenous administration and was compared with that in fluid aspirated from infected cysts.

**Results:**

In the patients undergoing cyst drainage, the mean serum MEPM concentration was 35.2 ± 12.2 μg/mL (range: 19.7 to 59.2 μg/mL, while the mean cyst fluid concentration of MEPM in the drained liver cysts (*n* = 12) or kidney cysts (*n* = 2) was 3.03 ± 2.6 μg/mL (range: 0 to 7.3 μg/mL). In addition, the mean cyst fluid/serum MEPM concentration ratio was 9.46 ± 7.19% (range: 0 to 18.8%). There was no relationship between the cyst fluid concentration of MEPM and the time until drainage after MEPM administration or between the cyst fluid/serum MEPM concentration ratio and the time until drainage.

**Conclusion:**

These findings suggest that MEPM shows poor penetration into infected cysts in ADPKD patients.

**Trial registration:**

This study was registered with the University Hospital Medical Information Network (UMIN) as “Penetration of meropenem into cysts in patients with autosomal dominant polycystic kidney disease (ADPKD)”, UMIN ID 000011292 on July 26th, 2013.

**Electronic supplementary material:**

The online version of this article (10.1186/s12882-018-1067-2) contains supplementary material, which is available to authorized users.

## Background

Autosomal dominant polycystic kidney disease (ADPKD) is the most frequent inherited kidney disease and is the fourth leading cause of end-stage renal disease (ESRD) among adults worldwide [1, 2]. Cyst infection is a frequent and serious complication of ADPKD, which sometimes becomes resistant to antibiotic therapy and can be fatal [3, 4]. While water-soluble antibiotics do not penetrate cysts well, lipid-soluble antibiotics show good penetration into cysts and are recommended for treatment of cyst infection in ADPKD [5]. However, we have increasingly encountered cyst infections that are resistant to lipid-soluble antibiotics. We recently investigated the bacterial pathogens causing cyst infection in ADPKD patients [4], and we found an unexpectedly high prevalence of bacteria that would be unlikely to respond to lipid-soluble antibiotics like fluoroquinolones. In addition, even gram-negative bacteria showed a high frequency of resistance to lipid-soluble antibiotics and some patients had cyst infection due to extended–spectrum beta-lactamase (ESBL)-positive gram-negative bacteria. Carbapenem therapy may be required for some cyst infections that are resistant to lipid-soluble antibiotics, especially in patients with ESBL-positive bacteria. Meropenem (MEPM) is a representative carbapenem with a broad spectrum of activity, but little is known about the penetration of such newer carbapenems into the cysts of ADPKD patients. Therefore, we investigated the penetration of MEPM into infected cysts of ADPKD patients to obtain data that could promote more appropriate and effective use of this antibiotic for cyst infection.

## Methods

This prospective observational study was reviewed and approved by the ethics committee of Toranomon Hospital in July 2013. This study was registered with the University Hospital Medical Information Network (UMIN) as “Penetration of meropenem into cysts in patients with autosomal dominant polycystic kidney disease (ADPKD)”, UMIN ID 000011292.

### Patients

We enrolled patients in the study according to the following criteria. All ADPKD patients receiving MEPM for cyst infection who underwent drainage of infected cysts at Toranomon Hospital from August 2013 to January 2014 were screened and patients without confirmation of definite cyst infection by detection of neutrophils in cyst fluid were excluded. We adopted the criteria for definite cyst infection of Sallee et al. (neutrophils in cyst fluid) [3] to ensure that only patients with definite infection were enrolled. However, our classification of hepatic cyst infection or renal cyst infection relied on the fact that the drained cysts contained neutrophils on cyst fluid analysis, and it did not ensure that the other organ had no concomitant infection. Therefore, there might be overlap between hepatic cyst infection, renal cyst infection, and infection in other organs. All 10 patients fitting these criteria gave written consent after being fully informed about this study.

### Clinical and laboratory findings

The symptoms of cyst infection were assessed from the clinical records, as were the laboratory findings using data obtained from the earliest tests performed after the onset of symptoms. The maximum body temperature (BT), the presence or absence of abdominal pain/back pain or tenderness and pyuria/haematuria, and the maximum white blood cell (WBC) count and serum C-reactive protein (CRP) level within one week after the onset of infection were recorded. The maximum BT, WBC count, and blood culture results were investigated before initiation of antibiotic therapy. These clinical data are presented in Table 1 and past medical history of each patient is presented in Additional file 1 (Past medical history of each patient).

**Table 1:** Clinical characteristics of all enrolled patients at the onset of cyst infection

Patient number	1	2	3	4	5	6	7	8	9	10	Total (mean ± SD)
Gender (M/F)	M	F	F	F	F	M	F	F	M	M	10 (4/6)
Age (years)	62	84	47	67	62	61	70	79	63	63	65.8 ± 8.9
Renal function	On dialysis	On dialysis	On dialysis	On dialysis	On dialysis	Serum Cr 0.88 mg/dL	On dialysis	On dialysis	On dialysis	On dialysis	Dialysis 9 Non dialysis 1
Body weight (kg)	61.4	42.7	51.5	51.1	41.4	59.9	47.1	48.1	55.8	66	52.5 ± 8.2
Total kidney volume (cm^3^)	4878.9	1696.1	1193.9	559.1	3429.6	8284.4	905.8	1386.0	1992.1	4498.7	2882.5 ± 2419
Liver volume (cm^3^)	5173.4	1527.9	4651.3	2903.0	2661.9	8965.7	2235.9	8340.5	4339.8	850.0	4164.9 ± 2734
Number of prior cyst infections	3	6	2	4	1	3	18	0	3	12	5.2 ± 5.6
Site of infected cyst	Liver	Liver	Liver	Liver	Liver	Liver	Liver	Liver	Kidney	Kidney	Liver 8 Kidney 2
Maximum body temperature (°C)	40.0	39.0	38.0	38.5	38.4	39.0	38.7	39.0	39.9	39.0	38.6 ± 1.3
Abdominal pain/flank pain/back pain	Flank pain	Epigastric pain	Right hypochondralgia	Epigastric pain	None	Epigastric pain	Epigastric pain	Right hypochondralgia	none	None	
Pyuria	None	None	None	None	None	None	None	None	None	None	
Blood culture test	*E.coli*	N	N	N	N	*E-coli*	N	N	*E-coli*	N	Negative 7
Cyst content culture test	N	N	N	N	N	N	N	N	N	N	Negative 10
Blood test											
Maximum WBC (/μL)	7050	6900	8500	11,400	7900	14,400	8800	8100	7100	7800	8795 ± 2358
Hb (g/dL)	10.0	9.6	10.5	7.7	10.1	8.6	9.4	9.8	9.6	11	9.6 ± 0.9
Total protein (g/dL)	8	4.9	7.4	6.9	7.3	8.2	6.9	7.7	7.4	6.6	7.1 ± 0.9
Alb (g/dL)	2.5	1.8	2.7	2.3	3.1	2.4	2.5	1.9	2.5	3.2	2.5 ± 0.4
GOT (IU/L)	14	7	15	9	16	29	18	17	19	12	15.6 ± 6.1
GPT (IU/L)	2	2	4	3	6	32	17	2	5	2	7.5 ± 9.7
ChE (IU/L)	122	192	272	245			158	60	214	199	195.5 (131, 237.3)^a^
ALP (IU/L)	392	277	199	290	613	720	499	273	359	260	324.5 (269, 527)^a^
γGTP (IU/L)	46	51	66	47	72	154	73	44	69	31	58.5 (45.5, 72.3)^a^
CRP (mg/dL)	21.84	20.90	21.20	8.9	21.4	16.0	26.9	10.7	15.50	12.8	18.5 (12.3, 21.5)^a^

*SD* standard deviation, *IQR* interquartile range (25–75%), *N* Negative, *NA* not applicable

^a^[median (IQR)]

### Imaging studies

Abdominal MRI was performed in all patients with suspected cyst infection in this study because none of them had contraindications to MRI such as a cardiac pacemaker. Abdominal computed tomography (CT) was performed in the patients with suspected cyst haemorrhage. When MRI was done, transverse and sagittal T1-weighted images (T1WI), T2-weighted images (T2WI), and diffusion–weighted images (DWI) were usually obtained, as reported previously [6]. None of the patients underwent gadolinium-enhanced MRI because 9 out of 10 patients had renal dysfunction. CT was performed as reported previously [6], and was usually done without enhancement because plain scans are adequate for identifying cyst haemorrhage and most of the patients had renal dysfunction. We did not perform 18-FDG PET/CT in any of the patients enrolled in this study.

### Selection and administration of antibiotic therapy

Our hospital policy was to only employ carbapenems in patients with cyst infection that was refractory to other antibiotics or patients with specific risk factors such as leukopenia, and physicians required submit reports to the infection control committee of Toranomon Hospital for using carbapenems. In all of the enrolled patients, the cyst infection had shown resistance to other antibiotics and treatment with MEPM was required. For administration, MEPM (0.5 g) was dissolved in 50 mL of saline and infused intravenously over 30 min. In each patient, we adjusted the MEPM dosage for renal function based on the Sanford Guide to Antimicrobial Therapy (2013). All patients on dialysis received the same dose of MEPM (0.5 g once a day) and the dose was not adjusted for body weight. The information about the antibiotics administered before MEPM and the duration of prior therapy, as well as the duration of MEPM administration in each patient are presented in the Additional file 2 (Antibiotics used in each patient).

### Aspiration of infected cysts

Percutaneous aspiration of an infected cyst was usually considered if a patient’s fever persisted for 1–2 weeks despite appropriate antimicrobial therapy as it is generally recommended [7]. Cyst drainage was performed on a non-dialysis day in all patients receiving hemodialysis. Infected cysts were detected according to our diagnostic criteria [6]. After the infected cyst was identified by abdominal MRI, aspiration was done under ultrasound guidance. A 10.2 Fr pigtail catheter with side holes was inserted percutaneously into each target cyst, and the contents were aspirated completely and submitted for culture. Some patients underwent aspiration of several infected cysts at the same time and the contents of each cyst were cultured. A drain was left in each cyst for one week after cyst fluid aspiration and daily lavage of the cyst cavity was performed with saline. We drained the cysts at various times after MEPM administration to investigate the relationship between the intracystic MEPM concentration and the time until drainage.

### Identification of bacteria

Identification of the isolates was performed using the MicroScan WalkAway 96 SI (Siemens Healthcare, Deerfield, IL, USA).

### Measurement of the MEPM concentration in serum and intracystic fluid

In all patients, a blood sample was collected just after completion of intravenous administration to measure the serum MEPM concentration. Most of the patients were on dialysis and had an arteriovenous fistula. Therefore, blood was collected from the wrist and antibiotics were administered into an antecubital vein of the same arm. The interval between MEPM administration and cyst puncture varied among the patients and the intracystic contents were sampled when a drain was placed into the infected cyst. Blood samples or cyst fluid samples were mixed with the same amount of 3-N-morpholino propane sulfonic acid buffer just after collection. The mixed samples were centrifuged for 10 min at 3000 rpm and then stored frozen at -80 °C. These samples were sent to the Department of Clinical Pharmaceutics, Faculty of Pharmaceutical Sciences, Doshisha Women’s College of Liberal Arts (Kyoto, Japan) in the frozen state, and all 300 μl of serum or cyst fluid was injected into an ultrafiltration device (Centrifree YM-30, Millipore) just after thawing. The MEPM concentration in plasma or cyst fluid was determined by injecting 30 μL of the filtrate obtained by centrifugation of the ultrafiltered sample at 3000 rpm for 5 min into a high-performance liquid chromatography (HPLC) system, which consisted of a liquid transport unit (LC-20AB, Shimadzu) and a spectrophotometer (SPD-20A, Shimadzu). The mobile phase was a mixture of PIC-A reagent/methanol (75:25), the flow rate was 1.0 mL/min, detection wavelength was 300 nm, and column was HYPERSIL ODS-5 (4.6 mm, L.D. × 250 mm; Chemco Scientific Co., Ltd.). For the assay using this system, the retention time of meropenem was 12.5 min, and lower limit of detection was 0.1 μg/mL. The intra-day and inter-day variation of MEPM measurement by this assay within 5% [8]. All measurements of MEPM were performed within 1 week after collection of samples from the patients. The residual rate of MEPM in plasma samples stored at -80 °C for 7 days after adding MOPS buffer was 99.5 ± 3.5% [8].

The MEPM concentration ratio between cyst fluid and serum was calculated as follows: cyst fluid MEPM concentration (μg/mL) / serum MEPM concentration (μg/mL) × 100 (%).

### Statistical analysis

Results are expressed as the mean ± SD for data analyzed by parametric tests and as the median with interquartile range for data analyzed by non-parametric tests. A probability (P) value of less than 0.05 was defined as indicating significance. Univariate regression analysis was performed to analyze the relationship between the cyst fluid MEPM concentration and serum MEPM concentration (MEPM concentration ratio), as well as that between the cyst fluid MEPM concentration and the time until drainage after MEPM administration, and that between the MEPM concentration ratio and the time until drainage after MEPM administration.

All statistical analyses were performed with the JMP® 13.0 statistical software package (SAS Institute Inc., North Carolina, USA).

## Results

Ten patients with 14 infected cysts were enrolled in this study (Table 1). The mean age of the patients was 65.8 ± 8.9 years. Nine patients were on dialysis and 8 patients had liver cyst infection. All of the patients showed elevation of CRP (median: 18.5; interquartile range [25–75%]: 12.3 to 21.5 mg/dL). Cyst fluid culture was not positive in any patient, but neutrophils were detected by cyst fluid analysis in all 10 patients, confirming the diagnosis of cyst infection. Blood culture was positive in 3 patients. The infection resolved within one month after cyst drainage in all 10 patients, and they were discharged from hospital.

**Fig. 1:**
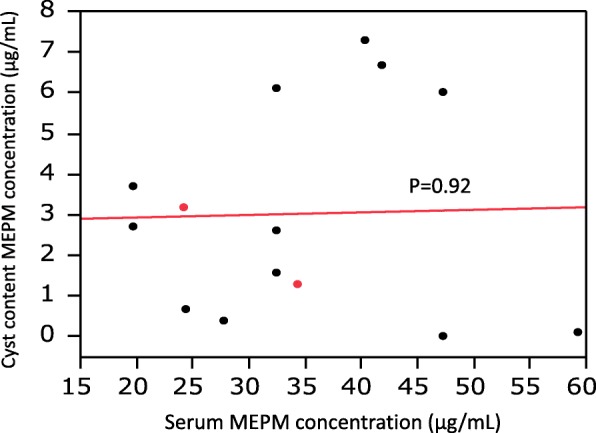
Relationship between the intracystic MEPM concentration and serum MEPM concentration. Black circles: patients with hepatic cyst infection, Red circles: patients with renal cyst infection

**Fig. 2:**
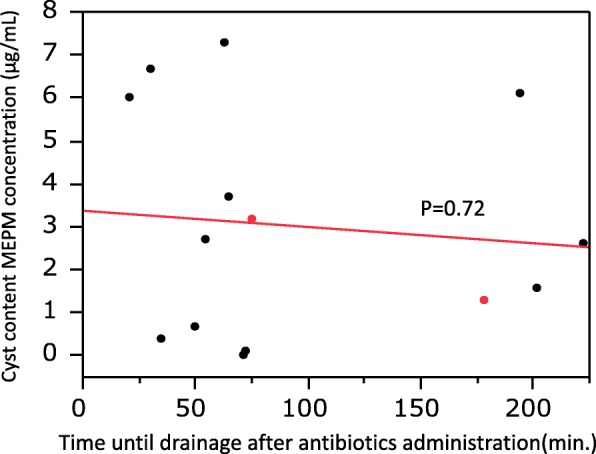
Relationship between the intracystic MEPM concentration and time to drainage after administration of MEPM. Black circles: patients with hepatic cyst infection, Red circles: patients with renal cyst infection

**Fig. 3:**
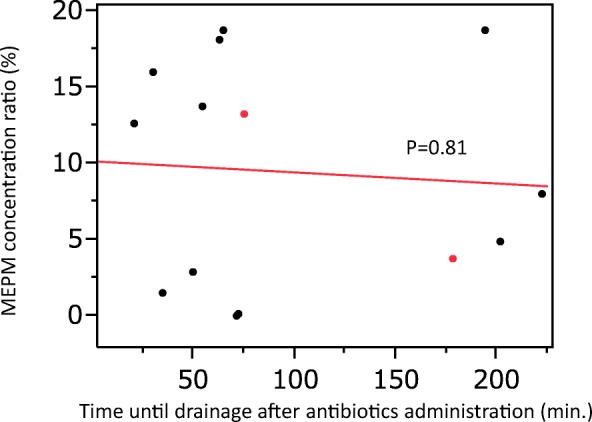
Relationship between the MEPM concentration ratio and time to drainage after administration of MEPM. Black circles: patients with hepatic cyst infection, Red circles: patients with renal cyst infection

Drainage of the infected cysts was performed between 21 and 222 min. after intravenous administration of MEPM (Table 2), with aspiration of 5–135 mL of the intracystic contents. The mean serum concentration of MEPM was 35.2 ± 12.2 μg/mL (range: 19.7 to 59.2 μg/mL), while the mean cyst fluid concentration of MEPM in the drained liver cysts (*n* = 12) or kidney cysts (*n* = 2) was 3.0 ± 2.6 μg/mL (range: 0 to 7.3 μg/mL). In addition, the mean MEPM concentration ratio was 9.46 ± 7.19% (range: 0 to 18.8%). There was no significant relationship between the cyst fluid MEPM concentration and the serum MEPM concentration (Fig. 1). There was also no significant relationship between the cyst fluid MEPM concentration and the time until cyst drainage after MEPM administration (Fig. 2), or between the MEPM concentration ratio and the interval from administration to cyst drainage (Fig. 3).

**Table 2:** MEPM concentration in serum and cyst fluid

Patient number	1	2	3	4	5	6		7		8			9	10	Total (mean ± SD)
Cyst number	1	2	3	4	5	6a	6b	7a	7b	8a	8b	8c	9	10	
Time between MEPM administration and cyst drainage (min.)	35	30	63	50	72	54	65	21	75	178	202	222	75	178	95.1 ± 70.5
Fluid volume aspirated from cysts (mL)	75	10	135	12	100	25	5	55	40	75	130	75	40	75	57.3 ± 44.0
MEPM serum concentration (μg/mL)	27.7	41.8	40.4	24.4	59.2	19.7		47.3		32.5			24.2	34.3	35.2 ± 12.2
MEPM cyst fluid concentration (μg/mL)	0.4	6.7	7.3	0.7	0.1	2.7	3.7	6.0	3.2	1.3	1.6	2.6	3.2	1.3	3.0 ± 2.6
MEPM concentration ratio (cyst content/serum) (%)	1.44	16.0	18.1	2.9	0.2	13.7	18.8	12.7	13.2	3.8	4.9	8	13.2	3.8	9.46 ± 7.2

## Discussion

It is often difficult to identify infected cysts in patients with ADPKD [6]. Therefore, we only enrolled patients whose cysts contained purulent fluid with abundant neutrophils, which confirmed that they had definite cyst infection. The serum MEPM concentration varied widely in this study, but this finding was consistent with previous reports that the Cmax of MEPM varied after a single intravenous dose of 0.5 g in patients on intermittent hemodialysis [9–13]. Our results suggested that MEPM shows poor penetration into infected cysts. MEPM is a water-soluble antibiotic, so this finding may be considered reasonable. However, MEPM is clinically effective for cyst infection in most ADPKD patients. One of the reasons for this apparent discrepancy might be that the minimum inhibitory concentration (MIC) of MEPM is relatively low for most bacteria. For example, the MIC_90_ of MEPM for *Escherichia coli* (*E-coli*) and *Klebsiella pneumonia* (typical gram negative bacteria) was only 0.03 μg/mL according to a survey performed in Japan [14]. However, the MIC_90_ of MEPM is high for some bacteria, e.g., 16 μg/mL for *Pseudomonas aeruginosa*. The intracystic MEPM concentration might not reach the effective level for such bacteria, which could explain why MEPM is not always effective for cyst infection. If cyst infection does not respond to antibiotic therapy with MEPM, we should consider that the intracystic MEPM concentration may be too low for it to be effective against the causative bacteria and we should also remember that *Enterococcus* spp. is frequent in patients with refractory cyst infection [4].

The cyst fluid/serum MEPM concentration ratio varied among the patients, but we could not identify any factors that influenced this ratio because of the small number of subjects enrolled in this study. Neither the cyst fluid MEPM concentration nor the MEPM concentration ratio was correlated with the time until cyst drainage after administration of MEPM, suggesting that the intracystic MEPM concentration might be maintained for a considerable period. Nine of the 10 patients enrolled in this study were on hemodialysis. In these patients, the serum MEPM concentration might have been maintained at a higher level because of decreased urinary excretion [9–13], which could have led to prolongation of higher intracystic MEPM concentrations. For β-lactam antibiotics, it is generally accepted that the bactericidal effect of these agents is determined by the time that concentrations of antibiotics are above the MIC (T > MICs) for the pathogens [15, 16]. This is another possible explanation for the effectiveness of MEPM (β-lactam antibiotics) for all patients in this study, despite showing poor penetration into the infected cysts.

Among the limitations of this study, the sample size was small and all of the patients were Japanese. In addition, 9 of the 10 patients were on dialysis and 8 patients had liver cyst infection. Therefore, our findings may not be generalizable to non-dialysis patients or patients with renal cyst infection. Furthermore, we only measured the serum and cyst fluid MEPM concentrations at one point in each patient, so we do not have information about the systemic MEPM concentration profile or its concentration in other cysts. We did not measure the serum of concentration MEPM at the time of cyst drainage, which might have led to underestimation of the MEPM concentration ratio (cyst content / serum). Further investigation will be needed to clarify the penetration of MEPM into infected cysts in patients with ADPKD.

## Conclusion

The findings of this study suggested that MEPM showed poor penetration into infected cysts in ADPKD patients. However, the cyst fluid MEPM concentration was higher than MIC_90_ of MEPM for typical gram negative bacteria (*E-coli* and *Klebsiella pneumonia*).

## Additional files

Additional file 1: Past medical history of each patient. (DOC 48 kb)

Additional file 2: Antibiotics used in each patient. (DOC 50 kb)

## Abbreviations

ADPKD: Autosomal dominant polycystic kidney disease
BT: Body temperature
CRP: C-reactive protein
CT: Computed tomography
DWI: Diffusion- weighted images
*E-coli*: *Escherichia coli*
ESBL: Extended–spectrum beta-lactamase
ESRD: End-stage renal disease
MEPM: Meropenem
MIC: Minimum inhibitory concentration
MRI: Magnetic resonance imaging
T1WI: T1-weighted images
T2WI: T2-weighted images
UMIN: University Hospital Medical Information Network
WBC: White blood cell
