# Supplementary material for: Cyst infection in autosomal dominant polycystic kidney disease: penetration of meropenem into infected cysts
Source: BMC Nephrol. 2018 Oct 19;19:272. doi: 10.1186/s12882-018-1067-2 (PMC6194587; doi:10.1186/s12882-018-1067-2)
Supplement: Supplementary file 1 — Past medical history of each patient. (DOC 48 kb) [file 12882_2018_1067_MOESM1_ESM.doc]

**Past medical history of each patient**

| Patient number | 1 | 2 | 3 | 4 | 5 | 6 | 7 | 8 | 9 | 10 |
| --- | --- | --- | --- | --- | --- | --- | --- | --- | --- | --- |
| Past medical history | np | Breast Cancer, Asthma | Chronic hepatitis B | chronic subdural hematoma | Jaw tumor | Cerebral hemorrhage | np | np | np | Cerebral hemorrhage, Asthma |

np: nothing particular
