# Supplementary material for: Cyst infection in autosomal dominant polycystic kidney disease: penetration of meropenem into infected cysts
Source: BMC Nephrol. 2018 Oct 19;19:272. doi: 10.1186/s12882-018-1067-2 (PMC6194587; doi:10.1186/s12882-018-1067-2)
Supplement: Supplementary file 2 — Antibiotics used in each patient. (DOC 50 kb) [file 12882_2018_1067_MOESM2_ESM.doc]

**Antibiotics used in each patient**

| Patient number | 1 | 2 | 3 | 4 | 5 | 6 | 7 | 8 | 9 | 10 |
| --- | --- | --- | --- | --- | --- | --- | --- | --- | --- | --- |
| Antibiotics administered before MEPM | LVFX+CLDM | Cefcapene pivoxil hydrochloride hydrate | LVFX | LVFX or CTRX or CFPM | Piperacillin sodium | Augmentin | LVFX | LVFX | Faropenem sodium | None |
| Duration of antibiotics therapy before MEPM (days) | 35 | 28 | 6 | 15 | 9 | 12 | 7 | 17 | 12 | NA |
| Duration of MEPM therapy before cyst drainage (days) | 16 | 12 | 5 | 7 | 34 | 8 | 5 | 8 | 8 | 62 |

NA: not applicable, LVFX: Levofloxacin, CLDM: Clindamycin, CFPM: Cefepime
